# Detection of two alphaviruses: Middelburg virus and Sindbis virus from enzootic amplification cycles in southwestern Uganda

**DOI:** 10.3389/fmicb.2024.1394661

**Published:** 2024-05-28

**Authors:** Selina Laura Graff, Georg Joachim Eibner, James Robert Ochieng, Terry C. Jones, Anthony Mutebi Nsubuga, Julius Julian Lutwama, Innocent Bidason Rwego, Sandra Junglen

**Affiliations:** ^1^Institute of Virology, Charité–Universitätsmedizin Berlin, Corporate Member of Freie Universität Berlin, Humboldt-Universität zu Berlin and Berlin Institute of Health, Berlin, Germany; ^2^Department of Zoology, Entomology and Fisheries Sciences, College of Natural Sciences, Makerere University, Kampala, Uganda; ^3^German Centre for Infection Research (DZIF), Partner Site Charité, Berlin, Germany; ^4^Centre for Pathogen Evolution, Department of Zoology, University of Cambridge, Cambridge, United Kingdom; ^5^Department of Plant Sciences, Microbiology and Biotechnology, Makerere University, Kampala, Uganda; ^6^Department of Arbovirology, Uganda Virus Research Institute (UVRI), Entebbe, Uganda; ^7^Department of Biosecurity, Ecosystems and Veterinary Public Health, Makerere University, Kampala, Uganda

**Keywords:** Middelburg virus, Sindbis virus, alphavirus, Togaviridae, arbovirus, mosquito, Uganda

## Abstract

Our knowledge of alphavirus genetic diversity is mainly based on viruses isolated from anthropophilic mosquito species, humans, and livestock during outbreaks. Studies on alphaviruses from sylvatic amplification cycles in sub-Saharan Africa have been conducted less often than from epizootic environments. To gain insight into alphavirus diversity in enzootic transmission cycles, we collected over 23,000 mosquitoes in lowland rainforest and savannah gallery forest in southwestern Uganda and tested them for alphavirus infections. We detected Sindbis virus (SINV) in a *Culex Culex* sp. mosquito and Middelburg virus (MIDV) in *Eretmapodites intermedius* and *Mansonia africana*. MIDV is a mosquito-borne alphavirus that causes febrile illness in sheep, goats, and horses and was previously not known to occur in Uganda. SINV, also a mosquito-borne alphavirus, causes mild infections in humans. Full genomes of SINV and MIDV were sequenced, showing a nucleotide identity of 99% to related strains. Both isolates replicated to high titres in a wide variety of vertebrate cells. Our data suggest endemic circulation of SINV and MIDV in Uganda.

## 1 Introduction

The genus *Alphavirus* in the family Togaviridae contains several mosquito-borne viruses that are maintained in sylvatic amplification cycles involving wildlife and forest-dwelling mosquitoes, such as Chikungunya virus (CHIKV) and Venezuelan equine encephalitis virus (Chen et al., 2018). While some viruses cause sporadic spill-over infections and local outbreaks in humans and domestic animals, others adapt efficiently to anthropophilic mosquito species and humans as amplification hosts, enabling spread to new geographic regions causing millions of infections worldwide, as is the case for CHIKV (Weaver et al., 1996; Weaver, 2014). According to the International Committee on Virus Taxonomy (ICTV), the genus *Alphavirus* contains more than 30 virus species, grouped into two monophyletic clades: (i) Old World alphaviruses, such as CHIKV, which can induce rash and chronic arthritis in humans, and (ii) New World alphaviruses, such as Eastern equine encephalitis virus, which can cause neurological disease in humans and horses with high mortality rates (Zacks and Paessler, 2010; Chen et al., 2018). Additionally, two aquatic alphaviruses and several insect-specific alphaviruses have been identified (Weston et al., 1999; Nasar et al., 2012).

Alphaviruses are characterized by small virions, typically 65–70 nm in diameter, and composed of a single capsid protein and three glycoproteins forming spherical, enveloped virions (Cheng et al., 1995; Chen et al., 2018). The genome of alphaviruses consists of 10–12 kb positive-sense, single-stranded ribonucleic acid (ssRNA), including two open reading frames (ORFs) (Chen et al., 2018). ORF1 codes for four non-structural proteins (nsP1-4) and ORF2 for six structural proteins (CP, E1-3, 6 K/TF) (Strauss and Strauss, 1994).

Sindbis virus (SINV), an Old World alphavirus, circulates among ornithophilic mosquitoes and birds in an enzootic transmission cycle (Lundström et al., 2019). SINV shares its main vector, mosquitoes of the genus *Culex*, with other emerging arboviruses such as West Nile virus (Mcintosh et al., 1976; Francy et al., 1989). Spill-over infections to humans and horses can occur via so-called bridge vectors, although they cannot sustain viral transmission (Lundström et al., 2019). Infection with SINV can cause an acute febrile illness with rash, arthritis, and musculoskeletal symptoms in humans (Skogh and Espmark, 1982; Espmark and Niklasson, 1984; Kurkela et al., 2005; Sem Ouilibona et al., 2018; Didier et al., 2024). SINV is geographically widespread and has been found in Africa, Europe, Asia, and Oceania, also designated as Babanki, Ockelbo, Karelian fever, Kyzylagach, or Sindbis-like virus, depending on the location of detection (Taylor and Hurlbut, 1953; Lundström and Pfeffer, 2010; Adouchief et al., 2016). It was first detected in Egypt in 1952. A total of six SINV genotypes were identified based on E2 gene phylogenetic analyses (Lundström and Pfeffer, 2010; Pickering et al., 2019). In Northern Europe, where the virus was most likely introduced by migratory birds from Central Africa in the 1920s, SINV is endemic and causes disease in humans (Turunen et al., 1998; Ling et al., 2019). While most studies on SINV have been conducted in Finland, Sweden, and South Africa, knowledge regarding its geographic distribution and incidence in other regions in Africa remains limited.

Middelburg virus (MIDV), another Old World alphavirus, was first isolated from *Aedes caballus* and *Aedes* (*Banksinella*) sp. mosquitoes in South Africa in 1957 and has subsequently been detected in Zimbabwe, Cameroon, Kenya, Senegal, and the Central African Republic (Kokernot et al., 1957; Attoui et al., 2007; Tricou et al., 2014). In southern Africa, MIDV has been found in livestock and wildlife, causing fever and encephalitis, but has not been associated with larger outbreaks (van Niekerk et al., 2015; Steyn et al., 2020).

Uganda is a hotspot region for arbovirus circulation and emergence, including several arboviruses that have been detected for the first time in Uganda’s forests, such as West Nile virus and Zika virus (Smithburn et al., 1940; Dick et al., 1952). This study aimed to sample mosquitoes from pristine and remote areas in Uganda that have an extraordinary amount of biodiversity (Convention on Biological Diversity, 2016). The objective was to assess the genetic diversity of alphaviruses in mosquitoes within enzootic amplification cycles in Uganda that have so far been neglected. Such explorations are crucial for detection and control of circulating (endemic) arboviruses, as well as to monitor genetic and phenotypic changes contributing to arbovirus emergence.

## 2 Materials and methods

### 2.1 Ethics statement

The Uganda Wildlife Authority (UWA) and the Uganda National Council for Science and Technology (UNCST) have approved a research permit for this project (number NS632).

### 2.2 Mosquito sampling

Mosquitoes were collected in southwestern Uganda in two different National Parks and the adjacent rural communities. Sampling was conducted in Maramagambo forest within Queen Elizabeth National Park (QENP), a gallery forest surrounded by savannah, and Semuliki forest within Semuliki National Park (SNP), the last lowland rainforest in East Africa, from April to October 2019. Sampling was carried out at three replicate sites within each National Park and the adjacent rural region, using 18 mosquito traps arranged in two parallel transects at each site. In the National Parks, four additional traps were positioned in the tree canopies at a height of 10–15 m to capture arboreal mosquitoes that feed on birds and monkeys. Three types of mosquito traps were used: (i) the BG Sentinel Trap (Biogents AG), baited with CO_2_ in combination with worn socks, 1-Octen-3-ol or BG-Lure, (ii) the CDC Light Trap, baited with a light source, and (iii) the CDC Gravid Trap (both John W. Hock Company), that mainly attracted gravid mosquitoes with a hay infusion. Traps operated for five consecutive days at each site. Mosquitoes were preserved in liquid nitrogen in the field. In the laboratory, mosquitoes were illuminated by a LED lamp while examined under a stereoscopic microscope (VWR SZT36P-Plus with 0,65 to 5,5 times magnification) for identification based on morphological criteria using classical identification keys and stored at −80°C (Edwards FWBM, 1941; Gillies, 1987; Jupp, 1996; Huang, 2004).

### 2.3 Screening for alphaviruses, molecular mosquito species identification, and testing for vertebrate blood-meal sources

Mosquitoes were homogenized individually in 500 μL phosphate-buffered saline (PBS) using ceramic beads and a Tissue Lyser II (QIAGEN) and then grouped into pools of 10 individuals by species, sex, and sampling site. RNA extraction was performed using a MagNA Pure 96 DNA and a Viral NA Small Volume Kit (Roche Diagnostics) on 140 μL of mosquito homogenate diluted with 60 μL of PBS, resulting in a 100 μL of RNA eluate. Subsequently cDNA synthesis was performed using SuperScript IV reverse transcriptase (Thermo Fisher Scientific) and random hexamer primers (Integrated DNA Technologies). The cDNA of eight mosquito pools was combined into one superpool and tested for alphaviruses using a generic Polymerase Chain Reaction (PCR) assay targeting the RNA-dependent RNA polymerase (RdRp) gene using Platinum® *Taq* polymerase (Life Technologies). Primers Alpha-F1 5′-TCAGCAGAAGAYTTYGAYGC-3′ and Alpha-R1 5′-CGTCCATGATYTTIACYTCCAT-3′ were applied for the first round PCR, and primers Alpha-F2 5′-CCTGTACTRGARACIGAYAT-3′ and Alpha-R2 5′-ACATTCCAGAYTTCATCAT-3′ were used for the nested PCR as described by Hermanns et al. (2017). The generated PCR products were separated and visualized using a QIAxcel DNA Screening Kit (QIAGEN) and sequenced by Sanger sequencing (Microsynth SeqLab). For PCR-positive superpools, the associated mosquito pools and individual mosquito samples were tested individually as described above. The generated nucleotide sequences were compared to the National Center for Biotechnology Information (NCBI) GenBank database using Geneious 2022.2.1 (Biomatters). Two PCRs based on the cytochrome c oxidase 1 (COI) gene were performed: first to confirm the mosquito species of virus-positive individuals, and second, to test for vertebrate DNA originating from a blood-feeding source (Folmer et al., 1994; Reeves et al., 2018). The nucleotide sequences generated were compared with the Barcode of Life Data System (BOLD) database (Ratnasingham and Hebert, 2007).

### 2.4 Virus isolation in cell culture and virus replication analyses

For primary isolation in cell culture, the supernatants of the PCR-positive mosquito pools were used. *Aedes albopictus* cells (C6/36), African green monkey kidney cells (VeroE6), and hamster kidney cells (BHK-21) were infected with a filtrated and an unfiltrated aliquot of the mosquito homogenate, the latter supplemented with an antibiotic (1% penicillin–streptomycin) and an antimycotic (1% Amphotericin B). The cells were examined daily for cytopathic effects (CPE). As soon as a clear CPE was visible or at the latest 7 days post-infection (dpi), the supernatant was harvested and used for passaging onto fresh cells. Aliquots of cell culture supernatants were used to test for virus replication using specific quantitative PCR (see below). Virus stocks (MP762-UG-2019: C6/36, filtrate, second passage, 2 dpi; MP61-UG-2019: BHK-21, filtrate, first passage, 3 dpi) were prepared in T-25 cell culture flasks, and the number of infectious particles was measured using a Tissue Culture Infectious Dose 50 (TCID50) end-point dilution assay (Reed and Muench, 1938; Marklewitz et al., 2013). Virus growth kinetics were performed in mosquito (C6/36), rodent (BHK-21), goat (ZNR), sheep (Llu-L), chicken (DF-1), blackbird (TME-R), non-human primate (Vero E6), and human (HEK) cells with a multiplicity of infection (MOI) of 0.1 and 0.01. All experiments were performed in duplicate. An aliquot of the cell culture supernatant was taken every 24 h for 4 days to measure the number of viral genome copies by quantitative real-time PCR using the primers and probes MIDV-F (5’-TCAGATTTCACTCCATGCACAATG-3′), MIDV-R (5’-ATGCTCAACATGACTATAGCTAGCA-3′), MIDV-TM (5’-CCGATAAAGGCGGCACAT-3′), SINV-F (5’-TTGAATGTCGTTATCGCCAGC-3′), SINV-R (5’-GTTGTCGTCGCCAATGAACG-3′), and SINV-TM (5’-AGCGGCTTAAAACGTCCAGA-3′).

### 2.5 Whole-genome sequencing

For viral genome sequencing, RNA was extracted from infectious cell culture supernatants using TRIzol (Life Technologies), followed by cDNA synthesis and library preparation using the KAPA RNA HyperPrep Kit (Roche). Subsequently, next-generation sequencing was performed using the MiSeq Reagent Kit v3 and a MiSeq desktop sequencer (Illumina). The generated reads were processed using an in-house pipeline and database. The 5′ and 3′ genome termini were amplified using 5’ RACE System for Rapid Amplification of cDNA Ends (ThermoFisher Scientific) and sequenced by Sanger sequencing.

### 2.6 Phylogenetic analyses

For phylogenetic analyses, nucleotide sequences of the structural polyprotein, the non-structural polyprotein, and the concatenated open reading frames (ORFs) of the structural and non-structural coding regions were aligned with sequences of related alphaviruses in Geneious 2022.2.1 using MAFFT (algorithm E-INS-i) (Katoh et al., 2002). The most appropriate substitution model for phylogenetic tree inference using the PhyML algorithm was identified in MEGA 11, resulting in the general time reversible (GTR) substitution model for SINV and the Tamura-Nei (TN93) substitution model for MIDV (Guindon and Gascuel, 2003; Guindon et al., 2010; Tamura et al., 2021). All phylogenies were computed using an estimated fraction of invariable sites and an estimated Gamma shape parameter. The bootstrap analysis was conducted based on 1,000 replicate trees.

### 2.7 Nucleotide sequence accession numbers

The complete genome sequences of one SINV strain and two MIDV strains have been registered in GenBank under accession numbers OR183436, OR183437, and OR183438, respectively.

## 3 Results

### 3.1 Detection of two alphaviruses in mosquitoes from primary ecosystems

A total of 23,473 mosquitoes was collected in Queen Elizabeth National Park (QENP, *n* = 2,718) and in Semuliki National Park (SNP, *n* = 20,755) in southwestern Uganda, morphologically identified, individually homogenized, and subsequently grouped into 312 superpools (SPs). SPs were tested for alphavirus infections using generic reverse transcription PCR. Alphavirus sequences were identified in three samples, SP8 from QENP, as well as SP97 and SP164 from SNP, all originating from undisturbed habitats inside the National Parks. Sequence fragments showed identities of 99.3% (SP97) to SINV and of 99.8% (SP8) and 100% (SP164) to MIDV. A single virus-positive mosquito was subsequently identified in each pool (Table 1). The morphologically identified mosquito species were confirmed by sequencing a fragment of the cytochrome c oxidase I (COI) gene indicating the presence of MIDV in female *Mansonia africana* (MP61-UG-2019) and *Eretmapodites intermedius* mosquitoes (MP1299-UG-2019) with 100 and 99.7% nucleotide identities, respectively. The COI gene sequence of the mosquito sample infected with SINV (MP762-UG-2019) showed a nucleotide identity of 97.5% to *Culex watti*. No vertebrate DNA was identified in the virus-positive individual mosquitoes by blood meal analysis.

**Table 1:** Sampling information of detected alphaviruses.

Sample ID	Virus	Strain	Mosquito species (Similarity COI in %)	Sampling site	Sampling date	Land-use type
SP8/M2168	Middelburg virus	MP61-UG-2019	*Mansonia africana* (100%)	Queen Elizabeth National Park	06/12/2019	Undisturbed gallery forest
SP97/M9848	Middelburg virus	MP1299-UG-2019	*Eretmapodites intermedius* (99.7%)	Semuliki National Park	08/19/2019	Undisturbed lowland rainforest
SP164/M7407	Sindbis virus	MP762-UG-2019	*Culex watti* (97.5%)	Semuliki National Park	09/22/2019	Undisturbed lowland rainforest

### 3.2 Virus isolation and growth analyses

**Figure 1:**
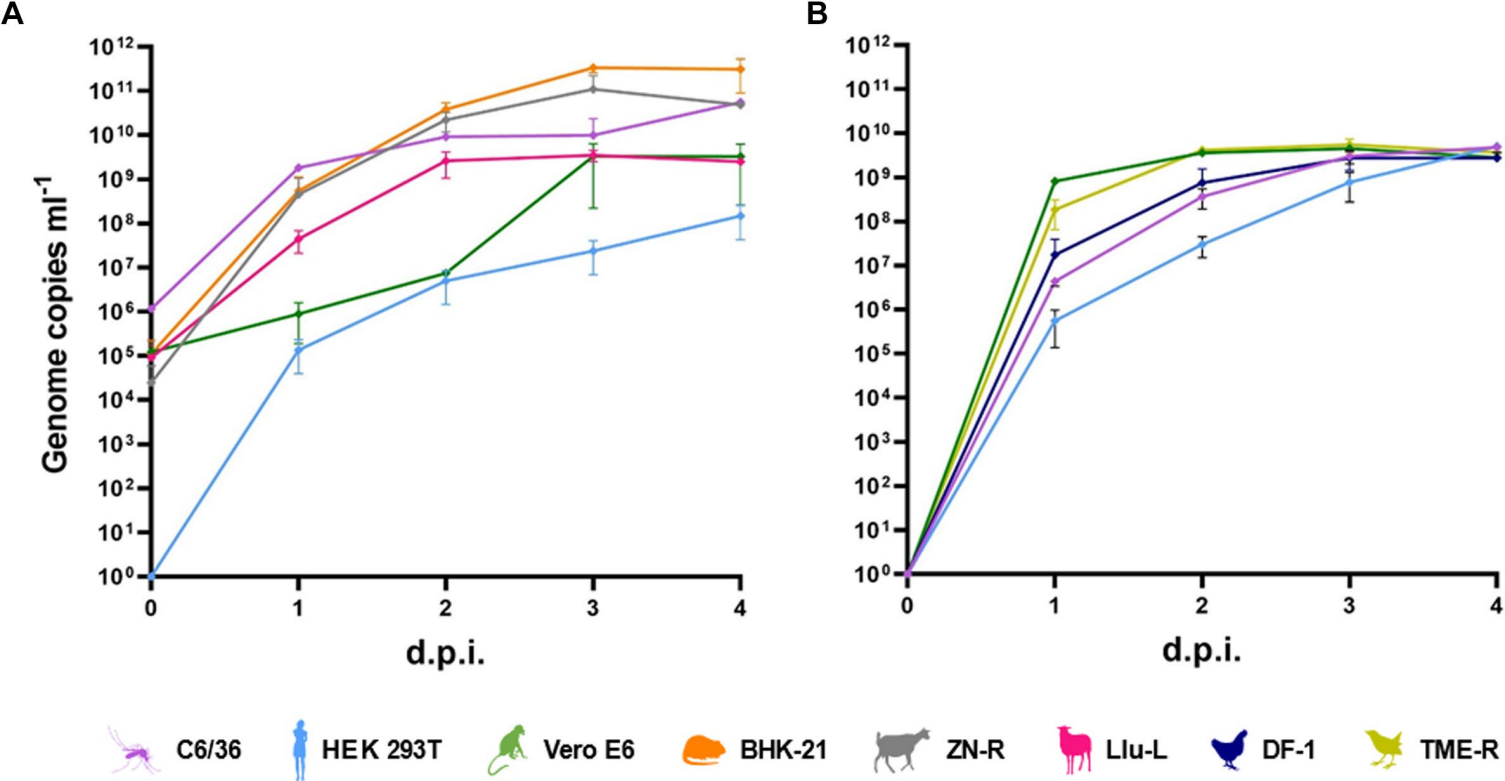
*In vitro* host range. **(A)** Growth kinetics of MIDV in mosquito (C6/36), human (HEK-293 T), monkey (Vero E6), rodent (BHK-21), goat (ZN-R) and sheep (Llu-L) cells infected with a multiplicity of infection of (m. o. i) of 0.01. **(B)** Growth kinetics of SINV in mosquito (C6/36), human (HEK-293 T), monkey (Vero E6), chicken (DF-I) and blackbird (TME-R) cells infected with a m. o. i. of 0.1.

SINV (MP762-UG-2019) and MIDV (MP61-UG2019, MP1299-UG-2019) were successfully isolated in cell culture. SINV MP762-UG-2019 and MIDV MP61-UG-2019 induced a clear cytopathic effect (CPE) 2 dpi in C6/36, VeroE6, and BHK21 cells, whereas MIDV MP1299-UG-2019 induced CPE 3 days after the first passage in C6/36 cells. Analyses of growth characteristics of SINV MP762-UG-2019 and MIDV MP61-UG-2019 showed that both viruses were able to infect a broad range of cell lines derived from various hosts, including mosquitoes, chicken, blackbird, rodents, goats, sheep, monkeys, and humans (Figures 1A,B). SINV MP762-UG-2019 showed rapid and successful replication in all tested cell lines reaching a plateau of approximately 10^9^ RNA genome copies/ml after 4 dpi. Genome copies of MIDV MP61-UG-2019 reached a plateau in all tested cell lines 3 dpi, albeit genome copies in C6/36, BHK-21, and ZNR cells were approximately 10–100-fold higher than in Llu-L and Vero E6 cells and approximately 1,000-fold lower in HEK293T cells.

### 3.3 Whole genome sequencing and genome analyses

The genomes of SINV MP762-UG-2019, MIDV MP61-UG-2019, and MIDV MP1299-UG-2019 were sequenced by next generation sequencing (NGS) using infectious cell culture supernatants. A high number of virus specific reads was obtained for each sample (MP762-UG-2019: 419.937 reads, MP61-UG-2019: 422.928 reads, MP1299-UG-2019: 340.805 reads) and allowed a gapless assembly of the coding regions. The minimum coverage for the coding regions of MP762-UG-2019, MP61-UG-2019, and MP1299-UG-2019 was 8,570, 4,267, and 1,614 reads, respectively. The mean coverage for the coding regions of MP762-UG-2019, MP61-UG-2019, and MP1299-UG-2019 was 14,389, 9,718, and 10,028 reads, respectively. The untranslated regions (UTR) at the 5′ and 3′ ends were determined by RACE-PCR. The coding regions showed the typical non-segmented genome organization of alphaviruses, including a read-through stop codon between non-structural protein (nsP) 3 and nsP4 genes (Strauss et al., 1983). Comparison of the structural and the non-structural polyprotein sequences of SINV MP762-UG-2019 to sequences deposited in Genbank using Basic Local Alignment Search Tool (BLAST) resulted in maximum nucleotide identities of 99.2 and 99.1% to a SINV isolate named Babanki virus (HM147984), respectively. The polyproteins of MIDV strains MP61-UG-2019 and MP1299-UG-2019 showed maximum nucleotide identities of 98.9% to MIDV ArTB-5290 that was isolated from *Amblyoma* var*iegatum* ticks in the Central African Republic in 1984 (KM115531), respectively. The high nucleotide identities of more than 98% in the coding regions confirm the detection of SINV and MIDV (Chen et al., 2018).

### 3.4 Phylogenetic relationship

**Figure 2:**
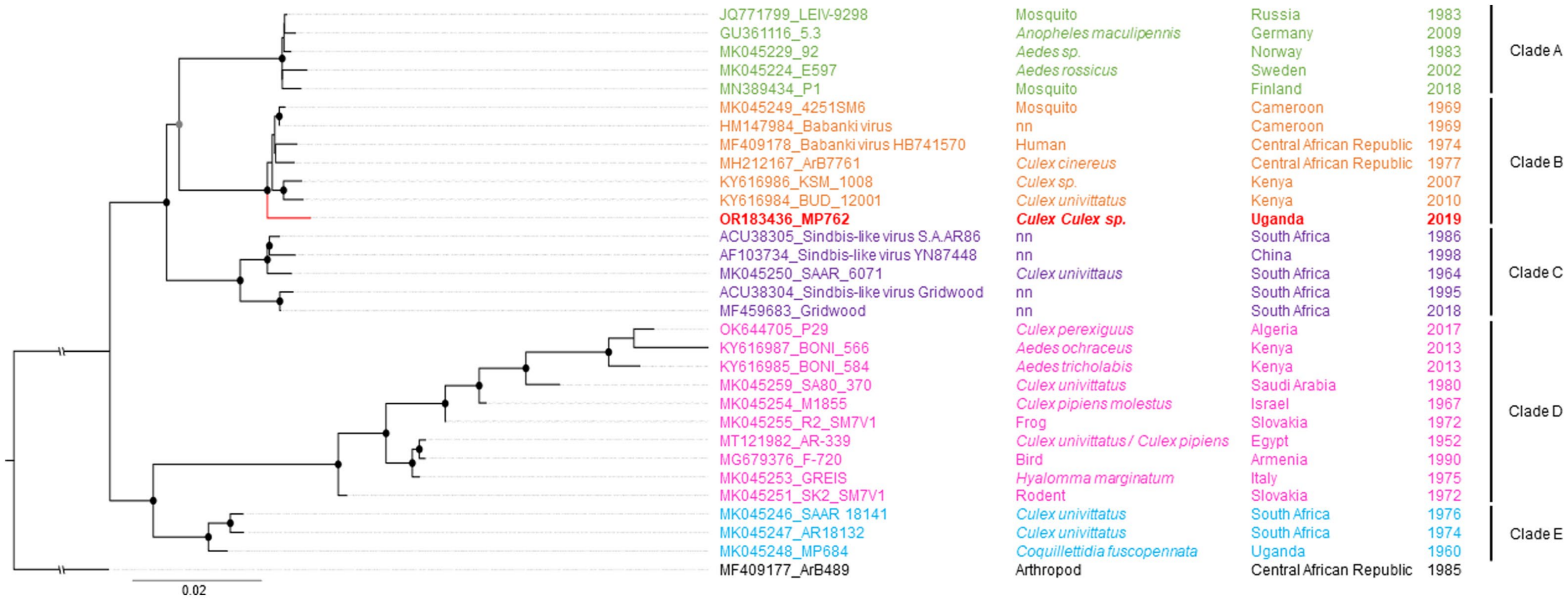
Phylogenetic relationship of SINV strain MP762-UG-2019. The Maximum likelihood (ML) phylogenetic tree of SINV-I is based on the concatenated nucleotide ORF sequences. The virus sequenced in this study is shown in red. SINV strains are labeled by GenBank Accession number, strain, source of isolation, country, and year of collection. The clades are named after Ling et al. (2019). Bootstrap support values are represented by gray (70–90%) or black (>90%) circles at the respective nodes.

Maximum likelihood (ML) analyses of the structural polyprotein and the non-structural polyprotein sequences, as well as the concatenated coding sequences of both ORFs of SINV MP762-UG-2019 and available SINV sequences, showed that the SINV strain detected in this study grouped within genotype I and was placed in a basal position in a clade with strains from Kenya (KY616984, KY616986), Central African Republic (MH212167, MF409178) and Cameroon (MK045249), termed clade B in Ling et al. (2019) (Figure 2). MP762-UG-2019 did not group with the only available other SINV sequence from Uganda (MK045248), detected in 1960 in *Coquillettidia fuscopennata* mosquitoes (formerly designated as *Mansonia fuscopennata*), which was placed in clade E. MP762-UG-2019 has a total of 78 unique nucleotide substitutions to other clade B sequences across the whole genome, of which 69 are synonymous and 9 are non-synonymous. One additional non-synonymous substitution is shared with two Kenyan strains (KY616984, KY616986). All non-synonymous substitutions were located in the non-structural polyprotein (Table 2).

**Table 2:** Non-synonymous substitutions and putative polyprotein position of MP762 to clade B viruses.

Putative gene	nt position	aa substitution
nsP1	1,219	T _387_ I
	1,381	S _441_ I*
nsP2	3,681	V _1,208_ I
nsP3	5,166	N _1,703_ D
	5,185	M _1,709_ T
	5,478	L _1,807_ F
	5,705	L _1,882_ F
nsP4	6,045	S _1,996_ T
	6,102	V _2,015_ I
	7,320	A _2,421_ S

*Shared with strains from Kenya (KY616984, KY616986).

**Figure 3:**
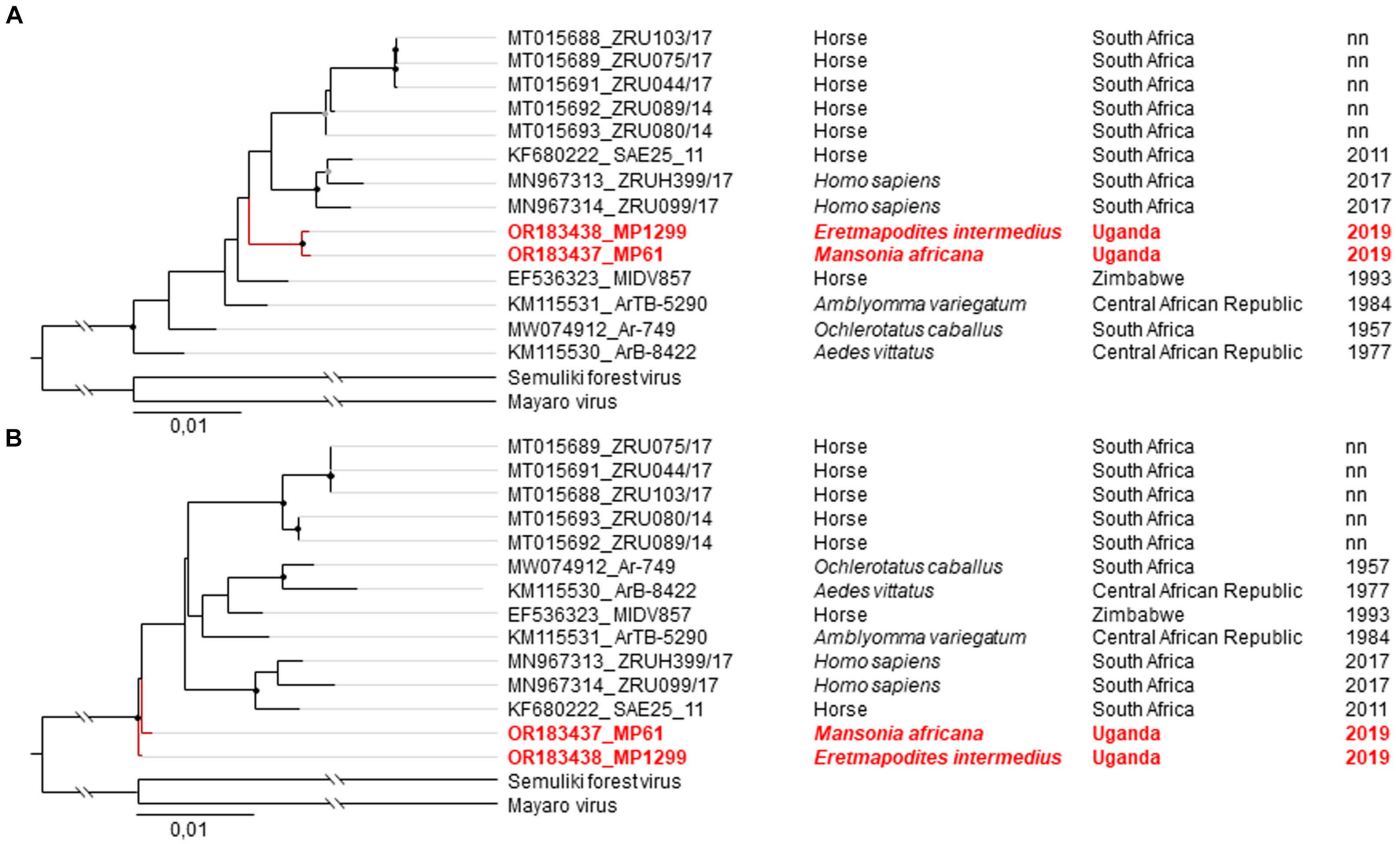
Phylogenetic relationship of MIDV strains MP61-UG-2019 and MP1299-UG-2019. The Maximum likelihood (ML) phylogenetic tree of MIDV based on the concatenated nucleotide ORF sequences **(A)** and the structural polyprotein **(B)**. The viruses sequenced in this study are shown in red. MIDV strains are labeled by GenBank Accession number, strain, source of isolation, country, and year of collection. Bootstrap support values are represented by gray (70–90%) or black (> 90%) circles at the respective nodes.

Phylogenetic analyses of the relationship of the two MIDV strains MP61-UG-2019 and MP1299-UG-2019 based on the non-structural polyprotein and the concatenated nucleotide ORF sequences placed both sequences as sister taxa in a clade with sequences from South Africa (MN967314, KF680222, MN967313) (Figure 3A). In the phylogenetic analysis based on the structural polyprotein sequences, the two MIDV sequences of this study did not group with any published MIDV sequence and were placed at the most basal position (Figure 3B).

MP61-UG-2019 and MP1299-UG-2019 both differed in 39 synonymous nucleotide substitutions from other MIDV sequences. MP61-UG-2019 showed eight additional synonymous substitutions to other MIDV sequences, including MP1299-UG-2019. MP1299-UG-2019 exhibits seven unique synonymous substitutions that distinguish it from other MIDV sequences, including MP61-UG-2019. Both sequences differ from each other by three non-synonymous nucleotide substitutions in the non-structural polyprotein and one in the structural polyprotein. The two sequences share one non-synonymous nucleotide substitution, which differentiates them from all previously published MIDV sequences (Table 3).

**Table 3:** Non-synonymous substitutions of MP61 and MP1299 compared to other MIDV sequences.

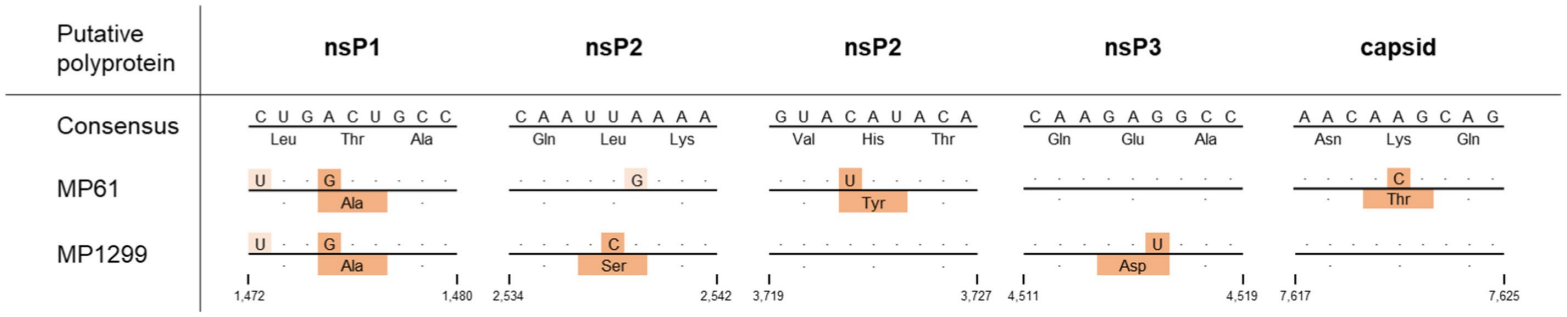	

## 4 Discussion

Arbovirus surveillance in hotspot regions of arbovirus emergence provides information on circulating strains and is important for correct diagnosis and disease control. Here, we collected over 23,000 mosquitoes in a lowland tropical rainforest, in a savannah gallery forest, and adjacent rural communities in southwestern Uganda and tested them for infection with alphaviruses. We detected SINV and MIDV in individual mosquitoes from two different primary habitat types, suggesting endemic circulation of these viruses in Uganda.

To our knowledge, this is the first detection of MIDV in Uganda. MIDV was found in Semuliki National Park and in Queen Elizabeth National Park. The presence of MIDV in two National Parks with distinct ecosystem types (lowland tropical rainforest and a savannah-like gallery forest) suggests endemic maintenance, and that MIDV has been circulating unnoticed for an unknown period in Uganda. So far, MIDV was associated with *Aedes* spp. mosquitoes as vectors (Jupp and Cornel, 1987). Here, we identified *Mansonia africana* and *Eretmapodites intermedius* mosquitoes as potential further MIDV vector species. Most mosquitoes of the genus *Eretmapodites* are sylvatic, while a few species have adapted to the disturbed environment in rural communities and breed in artificial containers such as the *Eretmapodites chrysogaster* group, which includes *Eretmapodites intermedius* (Someren, 1949; Lounibos, 1978). Birds and mammals serve as blood meal sources (Musa et al., 2020). *Mansonia* spp. mosquitoes are active at dawn and dusk, searching for a variety of hosts including birds, mammals, and reptiles (Galardo et al., 2022). Our understanding regarding host range and pathogenicity is limited. Experimentally infected sheep showed viremia and pyrexia, and infection of newborn mice was shown to be lethal (Kokernot et al., 1957). Horses can develop severe symptoms, including neurologic disease, sometimes with fatal outcome, as observed in Zimbabwe in 1993 and in South Africa in 2015 (Attoui et al., 2007; Van Niekerk et al., 2015). Antibodies against MIDV have been detected in humans and a recent study detected MIDV genome copies in cerebrospinal fluid and whole blood samples of patients with neurological symptoms suggesting that MIDV may cause disease in humans (Smithburn et al., 1959; Fourie et al., 2022). The efficient and rapid growth of the Ugandan MIDV isolate on the tested human and livestock cell lines suggests no overt impairment of the potentially enzootic isolate with respect to host range. However, future investigations aiming to identify MIDV in acute febrile patients and animals, as well as testing for the presence of neutralizing antibodies against MIDV in humans and animals, would be needed to shed light on the geographic distribution and burden of MIDV in Uganda.

The detection of SINV in a *Culex Culex* sp. mosquito from Semuliki National Park confirms previous findings of SINV in Uganda, where it was first detected in *Coquillettidia fuscopennata* mosquitoes in Buwaya near Entebbe in 1960 and shortly afterwards in humans in 1961 (Haddow, 1961/1962; Woodall et al., 1964). SINV has been found in *Culex* sp. and *Mansonia* sp. mosquitoes from Semuliki National Park and Lake Mburo National Park in 2010 (Mossel et al., 2017). Furthermore, SINV neutralizing antibodies were detected in bats from Kampala, suggesting that the virus occurs across the country (Kading et al., 2018). Across sub-Saharan Africa, SINV has been found in various mosquito species, including *Culex quinquefasciatus* from Burkina Faso, *Culex cinereus* from the Central African Republic, and *Culex univittatus*, known as main vector for SINV in countries such as South Africa and Kenya (McIntosh and Jupp, 1979; Sigei et al., 2018). We could not unambiguously identify the vector species as the mosquito species could not be identified on a morphological basis and its COI gene sequence showed only a maximum relationship of 97.5% to *Culex watti*. *Culex watti* has not yet been described as a vector for SINV. Although SINV was isolated from an enzootic vector, it is likely that it can readily infect humans and equids, as supported by cell culture infection experiments and phylogenetic analyses. SINV MP762-UG-2019 demonstrated rapid replication rates across all tested cell lines and was shown to belong to genotype-I, that has been reported from Africa, Europe, and the Middle East and is associated with febrile illness (Skogh and Espmark, 1982; Espmark and Niklasson, 1984; Kurkela et al., 2005; Sem Ouilibona et al., 2018; Didier et al., 2024). The symptoms are usually self-limiting and clear spontaneously within a few weeks, but in approximately 25% of patients, the symptoms can persist for years (Niklasson et al., 1988; Kurkela et al., 2005). Outbreaks were mainly reported from northern Europe, mainly Sweden and Finland, and sporadically from South Africa (Espmark and Niklasson, 1984; Jupp and Meenehan, 1986; Suvanto et al., 2022). For example, the incidence of SINV in Finland peaked with 566 reported cases in 2021, resulting in the most extensive outbreak of a mosquito-borne disease in Europe that year (Suvanto et al., 2022). As is the case for MIDV, surveillance for and diagnosis of SINV infection is limited in Uganda, and knowledge of human infection rates is unknown.

Like Chikungunya virus (CHIKV), MIDV and SINV are categorized as Old World alphaviruses, traditionally associated with milder disease compared to New World alphaviruses like the equine encephalitis viruses. Eastern equine encephalitis virus, along with Venezuelan and Western equine encephalitis virus, can lead to severe neurological disease with high mortality rates in both horses and humans (Meyer et al., 1931; Deresiewicz et al., 1997). The characterization of Old World alphaviruses as less severe warrants re-evaluation, given that they also induce significant clinical manifestations in humans and animals. For instance, CHIKV is associated not only with chronic arthritis but also with neurological manifestations in humans (Rampal and Meena, 2007). SINV and MIDV have also been linked to neurological symptoms in horses and other mammals (Van Niekerk et al., 2015; Steyn et al., 2020).

We observed inconsistencies in the placement of MIDV MP61-UG-2019 and MP1299-UG-2019 in phylogenetic analyses. In phylogenetic analyses based on concatenated ORF sequences and the non-structural polyprotein, both MIDV strains were placed as sister taxa in a clade with sequences from South Africa (Figure 3A). Phylogenetic analysis based on the structural polyprotein placed MP61-UG-2019 and MP1299-UG-2019 in basal relationship to all other sequences (Figure 3B). This inconsistency may originate from a recombination event between Semliki Forest virus and Mayaro virus within the E1 gene, as observed previously (Attoui et al., 2007). The basal position and the detection of the two strains in mosquitoes from primary ecosystems support an origin from sylvatic (enzootic) amplification cycles. The exploration of genetic and phenotypic traits of sylvatic variants helps to identify changes in emerging epidemic variants, as documented for CHIKV and VEEV (Wang et al., 1999; Powers et al., 2000). Especially CHIKV demonstrated a high epidemic potential and how fast a new emerging epizootic variant can spread geographically. In the last 15 to 20 years CHIKV has spread to new territories all over the world and is responsible for millions of human infections (Weaver, 2014; Wahid et al., 2017). Similarly, SINV showed that a solitary dispersal event can result in the establishment of a virus in a new geographic region (Ling et al., 2019), given conditions such as the availability and abundance of vector, host, and reservoir under appropriate climate conditions. It has been suggested that SINV-I was introduced to Sweden from central Africa via migratory birds in the 1920s and subsequently spread to central and southern Europe in the 1960s and 1970s (Ling et al., 2019). Particularly biodiversity loss, changes in land use, and climate have been suggested to facilitate the spread of viruses to new geographic regions (Institute of Medicine Forum on Microbial T, 2008).

## 5 Conclusion

This study highlights the importance of arbovirus surveillance in hotspot regions of arbovirus emergence for understanding the genetic diversity of circulating arboviruses and for the implementation of respective diagnostics and disease control. We identified the endemic circulation of SINV and MIDV in Uganda, as well as provided the first detection of MIDV in the country. Both viruses infect humans and MIDV also causes disease in livestock. The potential impact of these viruses on human and animal health is currently unknown as no seroprevalence studies have been conducted in the region. Clinical data from patients with compatible symptoms are also not available. It is likely that MIDV and SINV infections are underdiagnosed. However, the geographic distribution of SINV and MIDV in Uganda would need to be investigated for more thorough risk assessments with respect to human and animal health. It may also be the case that both viruses are mainly restricted to the sylvatic environments in Uganda. The isolation of SINV and / or MIDV from epizootic amplification cycles in Uganda could add knowledge regarding virus adaption and evolution during emergence processes from sylvatic amplification cycles. This study further raises questions regarding the pathogenicity, host range and amplification cycles of these viruses, particularly of MIDV, which has been less studied.

## Data availability statement

The datasets presented in this study can be found in online repositories. The names of the repository/repositories and accession number(s) can be found in the article/supplementary material.

## Author contributions

SG: Data curation, Formal analysis, Investigation, Methodology, Software, Validation, Visualization, Writing – original draft, Writing – review & editing. GE: Investigation, Writing – review & editing. JO: Investigation, Writing – review & editing. TJ: Methodology, Writing – review & editing. AN: Methodology, Resources, Writing – review & editing. JL: Methodology, Resources, Writing – review & editing. IR: Methodology, Resources, Writing – review & editing. SJ: Conceptualization, Funding acquisition, Project administration, Resources, Supervision, Writing – review & editing.
