# Erratum: Detection of two alphaviruses: Middelburg virus and Sindbis virus from enzootic amplification cycles in southwestern Uganda

**DOI:** 10.3389/fmicb.2024.1469636

**Published:** 2024-08-20

**Authors:** Frontiers Production Office

**Affiliations:** Frontiers Media SA, Lausanne, Switzerland

**Keywords:** Middelburg virus, Sindbis virus, alphavirus, Togaviridae, arbovirus, mosquito, Uganda

Due to a production error, there was a mistake in Table 2 as published. In the third column, the aa substitutions in the first three rows are incorrectly formatted and the aa substitutions in the sixth and seventh row are each missing a letter. The corrected Table 2 appears below.

**Table 2:** Non-synonymous substitutions and putative polyprotein position of MP762 to clade B viruses.

**Putative gene**	**nt position**	**aa substitution**
nsP1	1,219	T _387_ I
	1,381	S _441_ I^*^
nsP2	3,681	V _1, 208_ I
nsP3	5,166	N _1, 703_ D
	5,185	M _1, 709_ T
	5,478	L _1, 807_ F
	5,705	L _1, 882_ F
nsP4	6,045	S _1, 996_ T
	6,102	V _2, 015_ I
	7,320	A _2, 421_ S

^*^Shared with strains from Kenya (KY616984, KY616986).

The publisher apologizes for this mistake. The original article has been updated.

